# Factors conditioning pain control and reduction in post-cesarean section parturients: a cross-sectional study

**DOI:** 10.1186/s12884-024-06579-9

**Published:** 2024-05-22

**Authors:** Anna Bogusława Pilewska-Kozak, Magdalena Dziurka, Agnieszka Bałanda –Bałdyga, Marta Joanna Monist, Ewelina Kopiel, Krzysztof Jurek, Anna Francesca Łęcka, Beata Dobrowolska

**Affiliations:** 1 Department of Obstetrics and Gynaecology Nursing, Chair of Obstetrics and Gynecology, Faculty of Health Sciences, Medical University in Lublin, Lublin, Poland; 2 Department of Holistic Care and Nursing Management, Faculty of Health Sciences, Medical University of Lublin, Lublin, Poland; 3 Integrated Medical Care Department, Medical Faculty, Collegium Medicum, Cardinal Stefan Wyszynski University in Warsaw, Warsaw, Poland; 4 2nd Chair and Clinic of Gynecology, Faculty of Medicine, Medical University in Lublin, Lublin, Poland; 5 The Neonatal Unit of the University Clinical Hospital, No. 1 in Lublin, Lublin, Poland; 6 Sociology of Culture, Religion and Social Participation Institute of Sociological Sciences, The John Paul II Catholic University of Lublin, Lublin, Poland; 7 Saint Lazarus Hospice, The Society of Friends to People in Disease, Cracow, Poland

**Keywords:** Cesarean section, Beliefs, Pain control, Pain coping strategies

## Abstract

**Background:**

Pain experienced by women in the perinatal period constitutes a complex and multifaceted phenomenon. The aim of the study was to assess conditions of pain locus of control and pain reduction in post-cesarean section parturients.

**Materials and methods:**

A cross-sectional quantitative study with convenience sampling was performed among 175 hospitalized post-cesarean section women in hospitals in Eastern Poland in accordance with the Strengthening the Reporting of Observational studies in Epidemiology (STROBE) statement. A self-design questionnaire regarding general information and obstetrics/gynaecology medical interview, The Pain Coping Strategies Questionnaire (CSQ) and The Beliefs about Pain Control Questionnaire (BPCQ) were used. The inclusion criteria were as follows (1) age of ⩾18 years old; (2) cesarean section (CS); (3) period from the 13th hour to the end of the 72nd hour after the procedure; and (4) informed consent. The data was analyzed with IBM SPSS Statistics.

**Results:**

Internal locus of control (M = 14.02) was provided the highest value by the parturients and followed by chance events (M = 12.61) and doctors’ power (M = 12.18). Dominant coping with pain strategies in the post-cesarean parturients were coping self-statements (M = 19.06), praying or hoping (M = 18.86). The parturients assessed their pain coping (M = 3.31) strategies along with pain reduction (M = 3.35) at the moderate level. Higher pain control was correlated with cognitive pain coping strategies (β = 0.305; t = 4.632; *p* < 0.001), internal pain control β = 0.191; t = 2.894; *p* = 0.004), cesarean section planning (β = -0.240; t = -3.496; *p* = 0.001) and past medical history of CS (β = 0.240; t = 3.481; *p* = 0.001). The skill of reduction of pain was positively associated with cognitive pain coping strategies (β = 0.266; t = 3.665; *p* < 0.001) and being in subsequent pregnancy (β = 0.147; t = 2.022; *p* = 0.045). Catastrophizing and hoping were related to lower competences of coping with pain (B = − 0.033, SE = 0.012, β = − 0.206, T = -2.861).

**Conclusions:**

The study allowed for identification and better comprehension of factors conditioning pain control and pain reduction in parturients after the cesarean section. Furthermore, a stronger belief that pain can be dealt with is found in the parturients characterized by cognitive pain coping strategies and internal pain locus of control. The skill of reduction of pain is related to cognitive coping strategy and procreation status.

**Supplementary Information:**

The online version contains supplementary material available at 10.1186/s12884-024-06579-9.

## Background

Pain according to Revised International Association for the Study of Pain (IASP) force definition (2020, p. 1976–1982) is “an unpleasant emotional and sensory experience for human beings that is or can be related to potential or actual tissue damage which is affected by psychological, social and biological factors” [1]. In healthcare, pain should be constantly assessed and monitored as well as respected if reported by patients. Fulfilling an adaptive role, pain can influence psycho-social well-being of people. It can be manifested by both verbal and non-verbal signs. However, lack of verbal communication of pain should be remembered not to exclude experiencing of pain [1]. Pain that occurs in labour is a particular type of pain since it constitutes a complex and multifaceted phenomenon. An increase in pain intensity aids the physiologic process of labour progress [2, 3]. Giving birth is an exhilarating event for mothers that even though they experience pain they describe it as both a tearing apart or excruciating and pleasing feeling [4]. A lot of different factors have an impact on severity of pain. Psychological factors are one of them and they comprise a competence of using pain reduction techniques by patients as well as their beliefs about pain control [5].

Locus of control is a factor associated with individual perception of pain by patients and their ability of dealing with pain [6]. Individuals with internal pain locus of control are responsible and believe that they control pain and interpret their experiences as a result of activities undertaken by them. However, individuals directed at others, namely with external locus of control feel that they are less responsible and thus they usually refer more to external factors such as other people’s activities, luckiness or chance [7]. Dependence between health locus of control and health behaviours have been indicated [6]. Internal locus of control is related to more common health promoting behaviors undertaken due to individuals’ beliefs about their influence on the course of disease; thus increasing their sense of efficiency in this way [8].

In turn, coping strategies are defined as individuals’ attempts (cognitive and behavioral) aimed at establishing control and dealing with the situation perceived by the individuals as threat, to some extent, in the emotional and physical aspect [9]. According to Hamilton et al. [10] emotions play the main role in coping with pain (energizing force in self-regulation).

Caesarean section (CS) in 2020 in Europe was performed at least 1.12 million times [11]. One out of five (21%) of deliveries worldwide are completed with a C-Sect. [12]. Moreover, the global rates of CS have significantly increased. The World Health Organisation (WHO) predicts that it will grow to 29% till 2030 [12]. Countries in which there are more cesarean sections than normal vaginal birth deliveries are as follows Brazil, Cyprus, the Dominican Republic, Egypt and Turkey. A high percentage of CSs also remains in Romania (44.1%), Bulgaria (43.1%), Poland (39.3%), Hungary (37.3%) and the USA (3.2%) [12–15]. To compare the lowest percentage of CSs performed is found in France (19.7%), Lithuania and Estonia (19.4%), Sweden (16.6%), and Finland (16.5%) [12, 14].

An essential measure taken by the WHO in 2018 was the issue of recommendations concerning non-clinical activities aimed at diminishing the number of unnecessary cesarean Sect. [15]. Therefore, a crucial element of perinatal care was highlighted as the focus on women’s education covered by: (1) child birth training workshops; (2) nurse-led applied relaxation training programme; (3) psychosocial couple-based prevention programme; (4) psychoeducation [15]. In the case of psychoeducation directed to women suffering from anxiety before labour and conducted by a midwife/therapist, it should encompass, among other things, information about anxiety, restlessness prior to labour, normalization of individual behaviours and responses during labour, stages and process of labour, hospital procedures and ways of anaesthetizing labour pain [15]. Midwives work regarding preparing women for the experience of pre-labour anxiety provides clinical short- and long-term benefits. Those women who had anxiety prior to labour and had participated in psychoeducation on the subject, they less frequently had stressful retrospections of labour and endeavoured to natural vaginal birth in the future [16]. Furthermore, participation in psychoeducation on pre-labour anxiety in nulliparous women was associated with a diminished number of symptoms of post-partum depression and better preparation for labour and maternity [17].

Therefore, the objective of the work was to assess conditioning of pain locus of control and pain reduction in hospitalized parturients after the cesarean section in the first days following delivery.

## Materials and methods

### Aim

Assessment of conditioning of pain locus of control and pain reduction in post-cesarean section (post-CS) parturients.

### Study design

A cross-sectional descriptive study was carried in hospitals in Eastern Poland. The study was conducted in accordance with the Strengthening the Reporting of Observational studies in Epidemiology (STROBE) statement, (see S1.File) [18].

### Study participants

Raosoft Sample Size Calculator was used to determine the sample size. For a confidence level of 0.90, a margin of error of 0.05, and a response distribution of 0.70, a sample size of 227 was required (based on 1.12 million cesarean section performed in Europe in 2020 as a population size indicator [11]). A convenience sample of 230 respondents in the puerperium hospitalized in maternity wards were asked to take part in the study. Consent to participation in the study was provided by 175 hospitalized post-CS parturients. The research was performed in two hospitals in city Lublin. The inclusion criteria were as follows (1) age of ⩾18 years old; (2) cesarean section; (3) period from the 13th hour after the surgical procedure to the end of the 72nd hour after the procedure; and (4) informed consent to take part in the research. Exclusion criteria: (1) lack of informed consent, (2) health status not allowing to give informed consent, (3) age below 18 years old, (4) period before 13th hour after CS, (5) 4th and subsequent postoperative day.

According to Nilson et al. [19] CS typically implies a hospital stay for 2–3 days. In order to obtain informed consent to participate in the study, the authors decided to include participants in the period from the 13th hour after the surgical procedure to the end of the 72nd hour after the procedure to allow uninterrupted skin-to-skin contact between mother and child in the first hours after CS. In addition, pain treatment is administered immediately after the caesarean section, which could affect the results of the investigation performed immediately after CS.

### Instruments

To collect data the following research instruments were utilized:

1. The authors’ own questionnaire comprising 38 questions. The author’ self-designed survey questions were developed on the basis of a literature review [20–22] carried out by the researchers. The questionnaire was pilot-tested with a small sample group to identify any ambiguities or unclear items. Feedback from this pilot test was used to refine and improve the questionnaire. The first part of the questionnaire included open-ended and closed-ended questions of one choice concerning sociodemographic data like age, body weight, height, marital status, place of residence, education, material situation, professional status, and a type of work performed. The second part constituted closed-ended questions of one or multiple choice, semi-open questions and open-ended questions. They regarded the obstetrics and gynaecology medical interview, a category of emergency CS, reasons for CS, knowledge on CS, a type of an aesthesia, the course of the first days in the puerperium – complaints of pain and other possible problems/complications, methods of alleviating pain and the condition of a newborn.
2. The Pain Coping Strategies Questionnaire (CSQ) compiled by Anne C. Rosenstiel and Frances J. Keefe from the University of Colorado Health Sciences Center in 1983, in the Polish adaptation by Zygfryd Juczyński [23]. It is used to assess pain coping strategies and their effectiveness in pain management. It is aimed at assessment of adults, patients and individuals complaining of pain, though completely healthy people can also be researched. The CSQ allows for prediction of adjustment to complaints of chronic pain. Moreover, it is used to assess individual competences of applying different strategies to alleviate pain and deal with it [23]. The questionnaire consists of 42 items describing ways of coping with pain and two questions provided to evaluate individual’s own skills of dealing with pain and relieving it. The statements are attributed to each of the seven pain coping strategies – six cognitive and one behavioural – strategy of increasing behavioural activity. Among the cognitive strategies the following ones are distinguished: diverting attention, reinterpreting pain sensations, coping self-statements, ignoring pain sensations, praying or hoping, and catastrophizing. The aforementioned strategies belong to the following three factors: cognitive coping, diverting attention and restructuring as well as catastrophizing and hoping. The respondents assessed frequency of their behaviours in relation to pain felt by means of the 7-point Likert scale ranging from 0 - never do that to 6 – always do that. To assess pain management, the following items were rated from 0 – no control/no ability to 6 – complete control/complete ability. Whereas the assessment of degree of pain decrease ranged from 0 - no ability to reduce pain to 6 – complete ability to reduce pain. In each category of pain coping strategies, the results obtained were added. The range of the result in each strategy was from 0 to 36 points. The higher the result, the more significant the way of dealing with pain [23]. Separate interpretation was performed for two questions concerning the degree of pain control and pain reduction. The result ranged 0–6 points, the higher the result, the greater the significance of individual competences of coping with pain and diminishing it. The structure of factors can also be used to interpret the questionnaire. The factors distinguished (cognitive coping, diverting attention and restructuring as well as catastrophizing and hoping) can have a relationship with basic styles of coping with stress such as problem-focused coping, escape coping and emotion-focused coping [23]. The questionnaire does not contain any norms of interpreting it; thus, the application of other research results seem to be useful for comparison. The internal consistency of the Polish version of the CSQ was estimated by means of Cronbach alpha for the entire questionnaire of 0.80, though for particular strategies it exceeded 0.80 apart from diverting attention (0.64) and increasing behavioural activity (0.63).
3. The Beliefs about Pain Control Questionnaire (BPCQ) compiled by Suzanne Skevingtonfrom the School of Social Sciences, University of Bath in 1990, in the Polish adaptation by Zygfryd Juczyński [8]. It refers to scales assessing health locus of control and is used to assess beliefs about pain control in adult individuals who are ill or healthy and complain of pain. The BPCQ can be applied supportively in the diagnosis and therapy of pain patients [8]. The questionnaire encompasses 13 items divided into 3 factors. The factors assess the intensity of individual beliefs about managing pain (internal factors), an influence or power of doctors and chance events. The respondents completed the questionnaire on their own assessing to what extent they agree with the statement given by means of the 6-point Likert scale of 1 - no, I completely disagree; 2 –I disagree; 3- I rather disagree; 4 – I rather agree; 5 – I agree; 6 – yes, I completely agree. The BPCQ results are depicted in three dimensions. For each dimension, the sum of the results is calculated according to the diagnostic key provided – particular items are attributed to each dimension of pain locus of control. Each dimension indicates power of beliefs in the individuals researched concerning the influence of internal factors (W), doctors (L) and chance events on pain (P). The range of points obtained regarding internal control is 5–30 and for the rest two dimensions 4–24. The higher the result, the more powerful the belief that pain is managed by the influence of a particular factor [8]. The internal consistency of the Polish version of the BPCQ was estimated by means of Cronbach alpha of 0.75 for the entire scale. For particular dimensions of pain locus of control, power of doctors (L) it was 0.86, internal pain locus of control (W) 0.82 and chance events (P) 0.58.

### Data collection

After permission for data collection from managers of hospitals, the researchers provided the questionnaires on the wards where inpatients stayed after caesarean section. The questionnaires were completed by the respondents after obtaining verbal informed consent to participate in the study and following instructions how to fill them out provided by researchers. Each paper version of the questionnaire included introduction with aim of the study, information of the study procedure and statement that filling in questionnaire is understood as giving consent to participate in the study, and that every respondent has right to resign from the study at any time during filling in questionnaire. The questionnaires filled in were collected and put into envelopes to be provided for individuals responsible for the research.

### Statistical analysis

The data was analyzed with IBM SPSS Statistics for Windows, version 27 (IBM Corp., Armonk, NY, USA). Multiple linear regression analysis (the stepwise method) was used to identify independent variables which predicted pain control and pain reduction. The assumptions of linearity and homogeneity of variance were checked using scatter plots and no heteroscedasticity/no clear pattern was found in the plots. Skewness was within ± 1. Multicollinearity was checked and the minimum and maximum variable inflation factor (VIF) were 1.009 and 1.158 for pain control and 1.003 and 1.016 for pain reduction, respectively, indicating that there was no risk of multicollinearity. A general F-test and adjusted R-square were performed. Standardized Beta coefficients (β) were calculated to assess the level of association and statistical significance in the multiple regression analysis.

To assess potential predictors of pain control in the post-CS parturients, multiple linear regression was conducted by means of the stepwise method by introducing sociodemographic variables (age, marital status, place of residence, education, occupational and financial situation), obstetric history variables (planning of the pregnancy, number of pregnancies, CS planning, number of CS, breastfeeding, post-CS complications, medical complications, preparation for care of the baby, obtaining information about CS, attending antenatal classes, being with the baby after the delivery), variables regarding coping with pain and variables concerning health locus of control. To assess potential predictors of pain reduction in the parturients, the same set of variables was applied as in the case of pain control.

The results obtained of the analysis were assumed to be statistically significant at *p* < 0.05.

### Ethical issues

The research was carried out following the approval of the Bioethics Committee of the Medical University of Lublin (KE-0254/114/2016) and in accordance with the Helsinki Declaration principles. The respondents were informed about their anonymity in the research, voluntary choice to participate or refuse to participate, aim of the study, course of data collection and their right of resigning from taking part in the study at any time. Verbal informed consent to participate in the study was obtained to avoid any signature from the respondents and protect their anonymity. Filled in questionnaires were put into separate envelops to ensure respondent’ privacy.

## Results

### Study participants

The study encompassed 175 post-CS parturients. The mean age of the respondents was 30 years old. The majority of the participants lived in the urban area (*n* = 118; 67%), were married (*n* = 142; 81.1%) and had higher education (*n* = 134; 76.6%). Over a half of the respondents (*n* = 96; 54.9%) had the caesarean section for the first time in the planned mode (*n* = 89; 51%). Table 1 depicts sociodemographic data of the participants researched and their obstetric history along with their newborns’ condition.

**Table 1:** Sociodemographic data of the participants, their obstetric history and newborn condition

Variable	M	SD	Min	Max	Q1	Me	Q3
Age	30.56	4.48	18.00	42.00	28.00	31.00	34.00
			N		%		
Place of residence	Urban area		118		67.4		
	Rural area		57		33.6		
Marital status	Married		142		81.1		
	In partner relationship		33		18.9		
Education	Higher		134		76.6		
	Secondary		36		20.6		
	Vocational		5		3.0		
Financial situation*	Very good		37		21.1		
	Good		116		66.3		
	Average		22		12.6		
Planning of the current pregnancy	Planned		137		78.3		
	Not planned		38		21.7		
Obstetric status	First pregnancy		71		40.6		
	Second pregnancy		63		36.0		
	Third and subsequent pregnancy		41		23.4		
No. of CSs	First		96		54.9		
	Second		67		38.2		
	Third or further		12		6.9		
CS mode	Emergency		86		49.0		
	Elective		89		51.0		
Condition of a newborn after delivery (according to the APGAR scale)	Good (8–10 points)		164		93.7		
	Moderate(5–7 points)		6		3.5		
	Mothers did not know		5		2.8		

M – Mean; SD – Standard Deviation; Min – Minimum, Max – Maximum, Me – Median ; Q1–1st quartile ; Q3–3rd quartile; * The participants asses their financial situation with the use of the 5-point Likert Scale based on their opinion (1 – very bad, 2 – bad, 3 – average, 4 – good, 5 – very good)

The majority of the respondents had post-operative wound pain (*n* = 171; 97.7%) and thus they needed the administration of analgesics (*n* = 168; 96.0%). Over a quarter of the participants had pain on micturition (*n* = 45; 25.7%), a total of 37 of them had pain on defecation (21.1%). Table 2 shows data on the experience of pain on the first day following the surgery and the use of analgesics by the parturients.

**Table 2:** Subjective pain experience in the first day after the surgery and application of analgesics in the parturients

Variable		*N*	%
Pain*	Of post-operative wound	171	97.7
	On micturition	45	25.7
	On defecation	37	21.1
Application of analgesics	Yes	168	96.0
	No	7	4.0

*the respondents could provide multiple answers

### Pain locus of control and strategies of coping with pain

The highest value was attributed to internal pain locus of control (M = 14.02) among the respondents. Whereas, power of doctors (M = 12.18) and chance events (M = 12.61) were given similar values (Table 3).

**Table 3:** Pain locus of control in the post-CS parturients

Variable		M	SD	Min	Max	Q1	Me	Q3	Range
Pain locus of control (the BPCQ)	Internal	14.02	4.12	5.00	28.00	11.00	14.00	16.00	5–30
	Power of doctors	12.18	3.73	4.00	23.00	10.00	12.00	14.00	4–24
	Chance events	12.61	4.11	4.00	23.00	10.00	12.00	16.00	4–24

M – Mean; SD – Standard Deviation; Min – Minimum, Max – Maximum, Me – Median; Q1–1st quartile ; Q3–3rd quartile

The dominant strategy of coping with pain in the post-CS parturients was coping self-statements (M = 19.06) and praying and hoping (M = 18.86) (Table 4). The lowest value was found in the domain of reinterpreting pain sensations (M = 7.51). Moreover, the respondents assessed their abilities to cope with pain (M = 3.31) and reduce pain (M = 3.35) at the moderate level. Table 4 indicates the in-depth characteristics of coping with pain strategies in the research group.

**Table 4:** Coping with pain strategies in the post-CS parturients

Variable		M	SD	Min	Max	Q1	Me	Q3	Range
Coping strategies	Diverting attention	12.89	7.63	0.00	32.00	7.00	12.00	18.00	0–36
	Reinterpreting pain sensations	7.51	7.07	0.00	30.00	2.00	6.00	12.00	0–36
	Catastrophizing	12.34	7.53	0.00	34.00	6.00	12.00	18.00	0–36
	Ignoring pain sensations	13.19	7.60	0.00	33.00	7.00	14.00	18.00	0–36
	Praying and hoping	18.86	7.75	0.00	36.00	14.00	20.00	25.00	0–36
	Coping self-statements	19.06	7.08	0.00	36.00	15.00	19.00	24.00	0–36
	Increasing behavioural activity	13.88	7.12	0.00	36.00	9.00	14.00	18.00	0–36
Pain control		3.31	1.11	0.00	6.00	3.00	3.00	4.00	0–6
Pain reduction		3.35	1.03	0.00	6.00	3.00	3.00	4.00	0–6

M – Mean; SD – Standard Deviation; Min – Minimum, Max – Maximum, Me – Median; Q1–1st quartile ; Q3–3rd quartile

### Predictors of pain management

In the case of pain control, the final model (F (5.168) = 15.284; *p* < 0.001) predicted 30% of the variance (Adj. R2 = 0.296).The model meets the criteria for homoscedasticity of variances, and the residuals are normally distributed. Table 5 presents the summary model for the pain control score due to the stepwise multiple regression analysis.

**Table 5:** Model summary for the pain control score: stepwise multiple regression analysis

Model	*R*	*R* ^2^	Adjusted *R*^2^	Change *R*^2^	F change	*p*
1	0.340	0.115	0.110	0.115	22.431	0.000
2	0.447	0.200	0.190	0.084	18.018	0.000
3	0.489	0.239	0.226	0.039	8.819	0.003
4	0.516	0.266	0.249	0.027	6.283	0.013
5	0.545	0.297	0.276	0.031	7.330	0.007

R – coefficient of correlation; R^2 −^ R-squared; coefficient of determination, Adjusted R^2^ – modified version of R-squared that accounts for the number of predictors in the model; F change test based on F statistic used to determine the significance of R square change

The model obtained includes 5 essential predictors: cognitive coping with pain (β = 0.305; t = 4.632; *p* < 0.001), catastrophizing and hoping (β = -0.266; t = -4.137; *p* < 0.001), internal pain locus of control (β = 0.191; t = 2.894; *p* = 0.004), planning CS (β = -0.240; t = -3.496; *p* = 0.001) and past medical history of CS (β = 0.240; t = 3.481; *p* = 0.001). A higher control of pain was associated with cognitive coping with pain, internal pain locus of control, CS planning, history of CS. Catastrophizing and hoping were correlated with a lower pain control. Table 6 shows multiple regression analysis predicting the control pain score (final model).

**Table 6:** Multiple regression analysis predicting the control pain score (final model)

						95% CI		VIF
	B	SE	Beta	t	*p*	Lower	Upper	
(Constant)	2.597	0.340		7.646	0.000	1.926	3.267	
Cognitive coping	0.056	0.012	0.305	4.632	0.000	0.032	0.079	1.063
Catastrophizing and hoping	− 0.046	0.011	− 0.266	-4.137	0.000	− 0.068	− 0.024	1.009
Internal pain locus of control	0.052	0.018	0.191	2.894	0.004	0.016	0.087	1.060
CS (planned/ unplanned)	− 0.532	0.152	− 0.240	-3.496	0.001	− 0.833	− 0.232	1.156
Caesarean section (first/subsequent)	0.533	0.153	0.240	3.481	0.001	0.231	0.836	1.158

B -unstandardized beta; SE – standard error for the unstandardized beta; β – standardized beta; t – test; VIF – variance inflation factor; CI – confidence interval

### Predictors of pain reduction

To investigate potential predictors of pain reduction in the respondents, the same set of variables was taken into account as in the case of pain control. The in-depth data on the issue are depicted in Tables 7 and 8.

The final model (F (3.170) = 7.684; *p* < 0.001) predicted 10% of variance (Adj. R2 = 0.104). The model meets the criteria for homoscedasticity of variances, and the residuals are normally distributed (Table 7).

**Table 7:** Model summary for the reduction control score: stepwise multiple regression analysis

Model	*R*	*R* ^2^	Adjusted *R*^2^	Change *R*^2^	F change	*p*
1	0.241	0.058	0.053	0.058	10.632	0.001
2	0.313	0.098	0.088	0.040	7.588	0.007
3	0.346	0.119	0.104	0.021	4.087	0.045

R – coefficient of correlation; R^2 −^ R-squared; coefficient of determination, Adjusted R^2^ – modified version of R-squared that accounts for the number of predictors in the model; F change test based on F statistic used to determine the significance of R square change

The model obtained includes three essential predictors: cognitive coping with pain (β = 0.266; t = 3.665; *p* < 0.001), catastrophizing and hoping (β = -0.206; t = -2.861; *p* < 0.001), being in subsequent pregnancy (β = 0.147; t = 2.022; *p* = 0.045). The ability to decrease pain was positively correlated with cognitive coping with pain and subsequent pregnancy. Catastrophizing and hoping was associated with lower competences to cope with pain (Table 8).

**Table 8:** Multiple regression analysis predicting the reduction pain score (final model)

						95% CI		VIF
	B	SE	Beta	T	*p*	Lower	Upper	
(Constant)	3.391	0.255		13.306	0.000	2.888	3.894	
Cognitive coping	0.045	0.012	0.266	3.665	0.000	0.021	0.069	1.016
Catastophizing and hoping	− 0.033	0.012	− 0.206	-2.861	0.005	− 0.056	− 0.010	1.003
First preganacy/ third and subsequent pregnancy	0.308	0.152	0.147	2.022	0.045	0.007	0.609	1.016

B - unstandardized beta; SE – standard error for the unstandardized beta; β – standardized beta; t – test; VIF – variance inflation factor; CI – confidence interval

The variables such as: occupation, breastfeeding, post-CS complications, medical complications, preparation for care of the baby, obtaining information about CS, attending antenatal classes, being with the baby after the delivery did not demonstrate a statistical correlation with pain control and pain reduction among respondents.

## Discussion

Both pain locus of control after delivery and coping with pain strategies are midwifery issues of great significance which require more attention because of constantly increasing percentage of CSs performed [12]. In women predictors of labour pain experience are associated with having a sense of self-efficacy during the previous labour [24], their tendency to catastrophize pain [25] and sensitivity to anxiety and restlessness [26].

The research shows the need for further investigations and comprehension of labour pain, ways of dealing with it, promoting natural vaginal delivery as well as having positive experience during labour [27]. Therefore, the objective of the work was to assess conditioning of pain locus of control and pain reduction in the post-CS parturients.

Firstly, the results obtained can be summarized in the following way the parturients attributed the highest value to internal pain locus of control, then chance events and last but not least power of doctors. Different results were reported by Czerw et al. [28] where oncological patients suffering from ovarian cancer, breast cancer and endometrial cancer attributed pain locus of control to power of doctors. However, this diversity can result from different characteristics of the research groups, specificity of the perinatal and post-operative periods as well as specificity of oncological treatment. The results suggest that preparation for labour and delivery in pregnant women is of great significance mainly due to pain locus of control after the caesarean section. The literature reports also confirms positive impact of psychoeducation on perinatal women in their further performance in the new reality following the delivery [16, 17].

Secondly, the dominant strategy of coping with pain found in the post-CS parturients was coping self-statements along with praying and hoping. Similar results were obtained in the research of oncological patients [28–30], coronary heart disease patients [31], patients with internal diseases [32] and females suffering from endometriosis [33]. Therefore, support of women after labour and delivery provided by healthcare professionals seems to be essential and it should include women’s needs and spirituality, for example women should be enabled to have a contact and talk with a priest, close relatives and friends in the postpartum period. The research indicates that patients expressed their needs of being cared for by medical professionals who are aware of their patients’ spiritual and religious needs [34]. Caesarean section as a surgery requires from the midwives to carefully monitor both infant and mother health condition [35]. Skin-to-skin contact after CS provides a number of possible positive outcomes for the woman [35]. Result of the study Salomończyk et al. (2022) showed a duration of the sin to skin contact after caesarean section last 1 to 5 min in 30.10% of the cases [35]. A crucial aspect of the appropriate care is also systematic pain assessment performed by nurses/midwives. Insufficient administration of analgesics may result in a high level of post-operative pain in post-CS parturients [36].

Thirdly, as far as coping with pain strategies are concerned, the lowest value was found in reinterpreting pain sensations. Moreover, the parturients assessed their competences of dealing with pain and relieving it moderately. Similar findings were provided in the case of patients with endometriosis who rarely used the strategies of reinterpreting pain sensations and ignoring pain [33]. In order to improve the results regarding post-CS parturients’ own competences of dealing with pain and relieving it, healthcare professionals should pay greater attention to the issue for instance during preparing women for labour and delivery. Such preparation would encompass encouragement to pregnant women to start education provided on antennal classes, participate in prenatal education on coping with pain along with non-pharmacological therapy in pain management, and such education would be delivered during appointments and check-ups with doctors/midwives [15].

Fourthly, the final model comprises five essential predictors of pain control such as cognitive coping with pain, catastrophizing and hoping, internal pain locus of control, CS planning and history of CS. The literature review highlights that self-confidence and positive attitude towards pain in patients contribute to diminishing the level of perceived pain and decreasing use of analgesics [37]. Women who had a high level of their self-efficacy in pain management had lower frequency of analgesics administration and asked for painkillers/anaesthesia at more advanced stages of labour [38]. In order to improve post-operative patients’ performance, healthcare professionals are encouraged to use cognitive and behavioural activities related to patients beliefs about pain management [39].

Furthermore, the research results obtained revealed that catastrophizing and hoping were correlated with lower pain control in the parturients researched. To sum up, the results suggest that past medical history of CS, CS planning and having knowledge of CS/perinatal management seem to be of great importance in raising awareness of pain in the parturients. Similar results were obtained by Tabriz et al. [40] where patients’ catastrophizing was associated with a lower level of pain control. Patients who are of the opinion that health is dependent on external factors such as faith, coincidence or other people have tendency to use passive strategies of coping with pain (e.g. catastrophizing) that are characterized by a feeling of helplessness and dependence on others [41]. In turn, the research indicates that individuals who have beliefs that they are able to control their health [41], experience lower intensity of pain [34] and use active strategies of coping with pain. Moreover, passive coping with pain strategies are more frequently found in patients with higher intensity of pain [42, 43], patients with worse mental health and disability [43].

Fifthly, the model obtained encompasses three crucial predictors that reduce pain, namely cognitive coping with pain, catastrophizing and hoping, procreation status The ability to alleviate pain was positively correlated with cognitive coping with pain and procreation status. Catastrophizing and hoping were associated with lower competences of coping with pain. Internal pain locus of control was related to being responsible for decisions and activities taken [44]. This approach has been indicated to decrease the level of experienced pain-related stress [44, 45]. Moreover, it increases pain tolerance, which improves patient-healthcare relationship and their collaboration, and abilities of coping with pain [44, 45]. Interestingly, Christiaens et al. (2010) presented that Dutch post-CS females having positive attitude towards labour pain and controlling their use of analgesics required rarer administration of analgesics [46]. In turn, in the case of women from Belgium, their negative attitude to labour and delivery constituted predisposing factor for more frequent administration of analgesics [46]. Waldenstrom et al. (1996) found that more severe labour pain was felt by females with negative attitude towards pain and more frequently they were anxious during labour. Lesser emotional suffering in women was caused by acceptance of pain, and lack of pain acceptance more frequently led to requirement for pain reduction [47]. Sak et al. (2016) suggest that appropriate psychological preparation concerning beliefs about pain control may be linked to improvement of healthcare outcomes for patients in the future [48]. The application of perinatal psychoeducation regarding cognitive strategies for women who tend to catastrophize may contribute to identification and better comprehension of their individual strategies of coping with pain [49]. Having negative beliefs in the perinatal period has been proven to increase frequency of experiencing anxiety, having obstetric complications and intensity of pain [50]. An interesting outcome of the study was presented by Tułacz et al. (2021) where only 11.8% of respondents did not feel fear during pregnancy or postpartum period [51]. Among the reasons for concern women mentioned fear of separation of mother and child after childbirth [51]. Kanadys et al. (2022) suggest there is a need to increase individualized holistic psychoprophylactic care to postpartum women in poor and average financial situation and to those who are learning or studying by promoting the concept of self-care preferably already in the period before the conception [52].

## Research limitations

The first research limitation can result from the fact that the research group represented solely Eastern Poland; thus, it cannot be representative for the entire population of post-CS women in Poland. The second research limitation can result from lack of pain severity assessment in the post-CS parturients by means of the pain assessment scale. Another research limitation constitutes the fact that a cross-sectional study was used with convenience sampling which may imply that the results of the study cannot be generalised. Therefore, no constructive conclusions can be drawn to indicate explicitly dependences between the particular variables. Additionally, although the research shows significant p-values in the stepwise multiple regression analysis on the model summary for the pain control score the R2s are considered as low. The results of our study demonstrates the need for further investigations and comprehension of this issue which requires a comparison of our findings with other authors’ results and implies a conservative interpretation of the study findings.

## Conclusions

The research allowed for identification and better comprehension of factors conditioning pain control and pain reduction in the post-CS parturients. The past obstetric medical history of CS and planning CS in advance affect a higher level of pain locus of control. Moreover, a stronger belief that pain can be managed is found in the respondents who are characterized by cognitive coping with pain strategies and internal pain locus of control. However, the ability to manage pain, namely reduce pain is related to cognitive pain coping strategies, and procreation status since women in the postpartum period in subsequent pregnancy are better at reducing pain. Post-CD women who are characterized by catastrophizing and hoping have weaker beliefs about their abilities to deal with pain and lower competencies to cope with pain.

## Implications

The research results obtained indicate the necessity for the application of an individual approach to women concerning coping with pain strategies after the cesarean section as well as their own pain locus of control during women’s psychoeducation conducted by midwives and other healthcare professionals, for instance on antenatal classes. Furthermore, the results obtained suggest further investigations regarding the subject, for example referring to the degree of pain experience. Medical professionals’ comprehension of determinants of pain perception and ways of coping with pain used by parturients can have a positive influence on the quality of post-operative care on maternity wards.

### Electronic supplementary material

Below is the link to the electronic supplementary material.

Supplementary Material 1

## Data Availability

All data generated and analysed during the current study are not publicly available due to protect participants’ privacy and confidentiality, but are available from the corresponding author on reasonable request.

## Abbreviations

CS: Cesarean section
IASP: International Association for the Study of Pain
WHO: World Health Organisation
Post-CS: Post-cesarean section
STROBE: Strengthening the Reporting of Observational studies in Epidemiology statement
CSQ: Pain Coping Strategies Questionnaire
BPCQ: Beliefs about Pain Control Questionnaire
